# Lysophosphatidic acid promotes endometrial decidualization in recurrent implantation failure patients by regulating LPAR6

**DOI:** 10.3389/fcell.2025.1652740

**Published:** 2025-10-22

**Authors:** Shanshan Wang, Yang Zhang, Xindong Zhang, Xinyu Cai, Min Hong

**Affiliations:** ^1^ Center for Reproductive Medicine and Obstetrics and Gynecology, Nanjing Drum Tower Hospital Clinical College of Nanjing University of Chinese Medicine, Nanjing, China; ^2^ Jiangsu Key Laboratory for Pharmacology and Safety Evaluation of Chinese Material Medical, School of Pharmacy, Nanjing University of Chinese Medicine, Nanjing, China; ^3^ Center for Reproductive Medicine and Obstetrics and Gynecology, Nanjing Drum Tower Hospital, Affiliated Hospital of Medical School, Nanjing University, Nanjing, China

**Keywords:** recurrent implantation failure, decidualization, metabolomics, LPA, LPAR6

## Abstract

**Background:**

Impaired decidualization is associated with recurrent implantation failure (RIF) and lysophosphatidic acid (LPA) is known to play an important role in decidua formation. However, the specific impact of LPA in endometrial decidualization during RIF remains unclear.

**Methods:**

Metabolomics analysis was performed to identify differentially expressed metabolites (DEMs) in RIF patients Expression of the LPA receptor subtypes, LPAR1-6, was detected in both GEO datasets and clinical endometrial samples. An *in vitro* decidualization model was established by treating human endometrial stromal cells (hESCs) with medroxyprogesterone acetate (MPA) and 8Br-cAMP. The functional roles of LPA and its receptors (LPAR1-6) during decidualization were further investigated via RT-qPCR, ELISA, immunofluorescence, CCK-8 proliferation assays, Western blotting, and immunohistochemistry.

**Results:**

LPA was identified as a pivotal metabolite in RIF. Among the LPA receptors, LPAR1 and LPAR6 were highly expressed during *in vitro* decidualization of hESCs. LPA treatment significantly increased the levels of insulin-like growth factor binding protein-1 (IGFBP1) and prolactin (PRL) and promoted cytoskeletal reorganization Inhibition of LPAR6-but not LPAR1-attenuated hESCs decidualization, as evidenced by reduced mRNA and protein levels of decidual markers and altered cellular morphology. CCK-8 assays revealed that neither LPA stimulation nor LPAR1-6 inhibition significantly affected hESC proliferation. Furthermore, LPAR6 blockade abolished the enhancing effects of LPA on IGFBP1 and PRL mRNA expression, as well as PRL protein secretion. These results suggest that LPAR6 plays a critical role in LPA-mediated regulation of decidualization.

**Conclusion:**

LPA plays a significant role in the decidualization process of hESCs by regulating LPAR6, rather than LPAR1, providing insights into potential therapeutic target for RIF.

## 1 Introduction

Infertility, a widespread public health issue affecting approximately 13% of couples globally, has garnered increasing concern within the medical community (Huang et al., 2023). Advances in assisted reproductive technology (ART) have allowed couples previously considered infertile to attain successful pregnancies, representing a major stride in infertility treatment. Nevertheless, recurrent implantation failure (RIF) remains a significant obstacle, substantially compromising the clinical pregnancy rate after embryo transfer through ART (Benkhalifa et al., 2022).

RIF remains a complex and poorly understood phenomenon with a multifactorial etiology, and even lacks an internationally accepted consensus definition (Franasiak et al., 2021). Accumulating evidence indicates that impaired decidualization represents a major cause of implantation failure (Ng et al., 2020; Peter Durairaj et al., 2017). Decidualization is a process wherein endometrial stromal fibroblasts transform into specialized secretory decidual cells, capable of producing markers such as IGFBP-1 and PRL, and providing the essential environment for embryo implantation and growth (Zhang et al., 2013). Thus, elucidating the mechanisms underlying decidualization offers a scientific basis for minimizing implantation failure.

Metabolomics presents a promising avenue for exploring the metabolic state of biosamples. In hormonal replacement therapy patients undergoing frozen embryo transfer cycles, a metabolomics analysis revealed distinct serum metabolite changes during endometrial transformation (Zheng et al., 2023). Harden et al. observed substantial differences in the metabolic profiles between decidualized and non-decidualized endometrium (Harden et al., 2021). RoyChoudhury et al. identified eight metabolites that were altered in RIF patients compared to women with successful implantation (RoyChoudhury et al., 2016). Fu et al. reported significant differences in vaginal metabolomes between patients with unexplained RIF and those who achieved pregnancy in the first frozen embryo transfer cycle (Fu et al., 2020). However, metabolomics studies focusing specifically on endometrial tissue from RIF patients remain relatively scarce.

In this study, for the first time, we employed metabolomic profiling to identify oleoyl-L-α-lysophosphatidic acid (oleoyl-LPA) as a potential metabolic marker in the endometrial tissue of RIF patients and hypothesized that it is involved in the decidualization process of human endometrial stromal cells (hESCs). This research further explored the roles of oleoyl-LPA and its receptors in endometrial decidualization, with the aim to provide a theoretical foundation and potential targets for novel therapeutic strategies against RIF.

## 2 Methods

### 2.1 Patients and tissue collection

The study was approved by the ethics committee of our hospital (2013-408081-01). Written informed consent was signed by each participant for the use of their samples.

RIF was defined as the failure to attain a clinical pregnancy following at least three consecutive embryo transfers with a cumulative transfer of more than four high-quality cleavage-stage embryos or more than two high-quality blastocysts (Coughlan et al., 2014). The control group comprised patients who underwent assisted reproductive treatment due to male infertility and successfully conceived after the first embryo transfer.

Individuals were excluded if they had any of the following conditions: repeated pregnancy loss (two or more biochemical pregnancies or two or more abortions); a history of adverse pregnancy; or any clear cause of embryo implantation failure, including, but not limited to, moderate-to-severe intrauterine adhesions, a thin endometrium (less than 7 mm before transformation), adenomyosis, endometriosis, uterine fibroids (submucosal fibroids, non-submucosal fibroids larger than 4.0 cm and/or endometrial compression), untreated hydrosalpinx, reproductive tract malformations, severe immune diseases, severe coagulation abnormalities, endocrine system diseases; karyotype anomalies in one or both partners; contraindications to pregnancy or assisted reproductive technology; infectious diseases, sexually transmitted diseases, or *mycoplasma* and/or *chlamydia*.

In this study, we enrolled 26 patients with RIF and 35 patients as controls. As summarized in Table 1, all endometrial samples from the RIF group were in the secretory phase. Among the 35 control samples, 5 were in the proliferative phase and 30 in the secretory phase, of which 4 were utilized for the isolation of primary hESCs.

**TABLE 1:** Clinical characteristics of women enrolled in the present study.

Variables	Control (n = 35)		RIF (n = 26)	*p-value*
	Proliferative phase	Secretory phase	Secretory phase	
	(n = 5)	(n = 30)	(n = 26)	
Age (years)	29.80 ± 2.77	28.73 ± 3.06	30.15 ± 4.16	0.328
BMI (kg/m^2^)	23.38 ± 4.74	23.38 ± 3.05	22.64 ± 2.61	0.646
Basal FSH (IU/L)	6.98 ± 0.56	7.42 ± 2.01	7.07 ± 2.61	0.822
Basal LH (IU/mL)	5.25 ± 1.08	4.55 ± 2.25	4.84 ± 2.15	0.75
Basal E2 (pmol/mL)	36.00 ± 4.47	38.62 ± 16.13	47.70 ± 38.92	0.425

RIF, recurrent implantation failure; BMI, body mass index; FSH, follicle stimulating hormone; LH, luteinizing hormone; E2, estradiol.

### 2.2 Metabolomics analysis

Metabolomics analysis was carried out on endometrial tissues from ten patients with RIF and ten fertile control patients. Endometrial tissue samples (100 mg) were homogenized in a high-throughput tissue grinder and suspended in pre-chilled 80% methanol. After placing in an ultrasonic cleaner for 10 min, the samples were centrifuged at 12,000 rpm for 10 min at 4 °C. The supernatants were filtered through a 0.22 µm filter and then injected into a LC-MS/MS system for analysis (Novogene Co., Ltd., Beijing, China). Differentially expressed metabolites (DEMs) were identified through partial least squares discriminant analysis (PLS-DA) using metaX, with screening criteria set as variable importance in projection (VIP) > 1, fold change (FC) > 1.2 or FC < 0.833, and *p* < 0.05. The resulting DEMs were visualized using the ggplot2 package in R.

### 2.3 Real-time quantitative PCR (RT-qPCR)

Total RNA was extracted from 10 mg endometrial tissues or cultured hESCs using TRIzol reagent (Vazyme, #R401-01) according to the manufacturer’s protocol. RNA integrity and concentration were assessed by agarose gel electrophoresis and spectrophotometry at 260/280 nm (One Drop, OD1000+). The extracted RNA was then reverse-transcribed into cDNA using the 5X All-In-One RT MasterMix (ABM, #ABS-G492). RT-qPCR was performed in a 20 µL reaction volume with ChamQ Universal SYBR qPCR Master Mix (Vazyme), using 18S rRNA as the endogenous control. The primer sequences are listed in Supplementary Table S1.

### 2.4 Isolation, culture and identification of primary hESCs

Fresh endometrial tissues were collected from fertile control women during the secretory phase. The endometrial tissues were minced and enzymatically digested with 0.1% (w/v) type I collagenase (Worthington, Freehold, NJ, United States) for 30 min at 37 °C. The stromal cells and glands were then separated by filtering the digested tissues through a 30 µm sieve. The isolated cells were resuspended in DMEM/F12 (Gibco, #10-092-CVRC) supplemented with 10% FBS (Gibco, #1645615) and 1% penicillin–streptomycin (Gibco, #SV30010), and then incubated at 37 °C with 5% CO_2_ until confluent. Stromal cell purity was verified by immunofluorescence assay for vimentin and E-cadherin as described below. Cells were cryopreserved in liquid nitrogen upon reaching 80%–90% confluence. No more than three passages were used for any cell strain. The hESCs were seeded into 60 mm or 35 mm culture dishes. At 90% confluence, the medium was replaced with serum-free DMEM/F12. After an overnight starvation period, cells were switched to phenol red-free DMEM/F12 containing 2.5% charcoal-stripped FBS. The hESCs were induced for 72 h and subsequently harvested for later experiments.

### 2.5 *In vitro* decidualization

The hESCs were incubated with 1 μM medroxyprogesterone acetate (MPA, Millipore Sigma, #M1629) and 0.5 mM 8Br-cAMP (Sigma #B7880) to generate an *in vitro* decidualization model, with medium replacement at 48 h. To determine the effect of estrogen (E2) and progesterone (P4) on LPAR1 and LPAR6 expression, hESCs were treated with E2 (10^−8^ M, Millipore Sigma, #E2758), P4 (10^−6^ M, Millipore Sigma, #P0130), E2+P4, and 8Br-cAMP + MPA in a time-dependent manner for 4 days. The cells and/or supernatants of hESCs were collected at different times for subsequent experiments.

### 2.6 The effect of oleoyl-LPA on decidualization

To elucidate the influence of oleoyl-LPA on decidualization, hESCs were treated with oleoyl-L-alpha-lysophosphatidic acid (Selleck #E2992) at concentrations of 0.1 μM and 1 μM. The hESCs and/or supernatants from different groups were harvested for subsequent experiments following 72 h of induction.

### 2.7 Enzyme-linked immunosorbent assays (ELISAs)

The PRL protein level in supernatants of hESCs after different treatments was determined by ELISA kit (Elabscience, #E-EL-H0141).

### 2.8 Immunofluorescence assay

The hESCs were prepared as described previously (Cai et al., 2022). Briefly, hESCs were fixed with 4% paraformaldehyde (Biosharp, #BL539A) for 20 min and permeabilized with 0.1% Triton X-100 (Sangon Biotech, 9002-93-1) at room temperature for 5 min. The hESCs were then blocked with 1% Triton X-100 containing 3% BSA for 30 min at 37 °C, incubated with primary antibodies (Supplementary Table S2) at 37 °C for 2 h, then with secondary antibody at 37 °C for 1 h. Nuclei were stained with DAPI. Cells were kept from light before being examined and digitally imaged with a fluorescence microscope and CCD camera.

### 2.9 Cell transfection

Primary hESCs at passages no higher than three were seeded into 6-well plates at a density of 2 × 10^5^ cells per well. At 60% confluence, the medium was replaced with serum-free and pen/strep-free basal medium. Cells were transfected with small interfering RNAs (siRNAs) targeting LPAR6 (siLPAR6-2/3) or negative control siRNA using Lipofectamine 3000 reagent (Invitrogen). After 6–8 h, the transfection medium was replaced with fresh complete culture medium. Decidualization was induced 48 h post-transfection, and total RNA and protein were harvested 72 h after induction from the respective experimental groups. All siRNAs for LPAR6 were synthesized and purified by Gene Pharma Co., Ltd. (Shanghai, China). The siRNA sequences were as follows:

siLPAR6-2:

sense: 5′-GGUGUUUGUGCUUGGGUUATT-3′,

antisense: 5′-UAACCCAAGCACAAACACCTT-3’;

siLPAR6-3:

sense: 5′-GCAUAACCUACAGACCUUATT-3′,

antisense: 5′-UAAGGUCUGUAGGUUAUGCTT-3’.

### 2.10 CCK-8 assay for cell viability and proliferation

Following resuscitation of cryopreserved 2^nd^-passage cells in DMEM/F12 (Gibco, #10-092-CVRC) plus 10% FBS (Gibco, #1645615) and 1% penicillin-streptomycin (Gibco, #SV30010), they were seeded into 96-well plates at a density of 5 × 10^3^. The cell were placed in an incubator with 5% CO_2_ at 37 °C for 24 h to ensure adequate cell adherence. After adhesion, the original medium was removed from the well, cells were transfected with siRNAs using Lipofectamine 3000 as mentioned before or treated with the following reagents: oleoyl-LPA, AM095 (4'-[3-methyl-4-((R)-1-phenyl-ethoxycarbonylamino)-isoxazol-5-yl]-biphenyl-4-yl-Na, MCE, HY-16040), and Ki16425 (3-(4-[4-([1-(2-chlorophenyl)ethoxy]carbonyl amino)-3-methyl-5-isoxazolyl] benzylsulfanyl) propanoic acid, Selleck #S1315) and incubated at 37 °C for 24 h. Lastly, CCK-8 reagent was added, the plates were incubated at RT for 20 min, and the absorbance at 450 nm was measured using a plate-reader.

### 2.11 Western blotting

Total protein was extracted as previously described (Cai et al., 2022). The proteins were separated by 10% SDS-PAGE and transferred to polyvinylidene difluoride membranes. After blocking, the membranes were incubated overnight at 4 °C with primary antibodies (Supplementary Table S2), followed by thorough washing and incubation with the corresponding secondary antibody for 1 h at RT. The band density was determined using ChemiCapture software (Beijing Sage Creation Science Co.).

### 2.12 Immunohistochemical staining

Endometrial tissues were obtained from endometrial biopsy and fixed in 4% PFA at room temperature. The tissues were dehydrated, embedded in paraffin and cut into 5 μm-thick sections. Antigen retrieval was conducted by autoclaving the samples at 121 °C for 15 min in the presence of a citrate antigen retrieval solution. Sections were blocked in 5% BSA, incubated overnight at 4 °C with primary antibody (Supplementary Table S2), washed and incubated with HRP-conjugated secondary antibody. The specific antibody signal was visualized by incubation with diaminobenzidine and counterstaining with hematoxylin. Images were captured using a Leica DM 2000 microscope.

### 2.13 Statistical analysis

Metabolomic data was processed with R (version 3.4.3). Graph Pad Prism 9.0 was used for statistical treatment of experimental data, and the quantitative data were presented as the mean ± SEM. Student’s t*-*test was used to assess significant differences between two groups, and ANOVA and Tukey’s test were used among multiple groups. The threshold for statistical significance was set at *p* < 0.05.

## 3 Results

### 3.1 Metabolomics analyses

**FIGURE 1:**
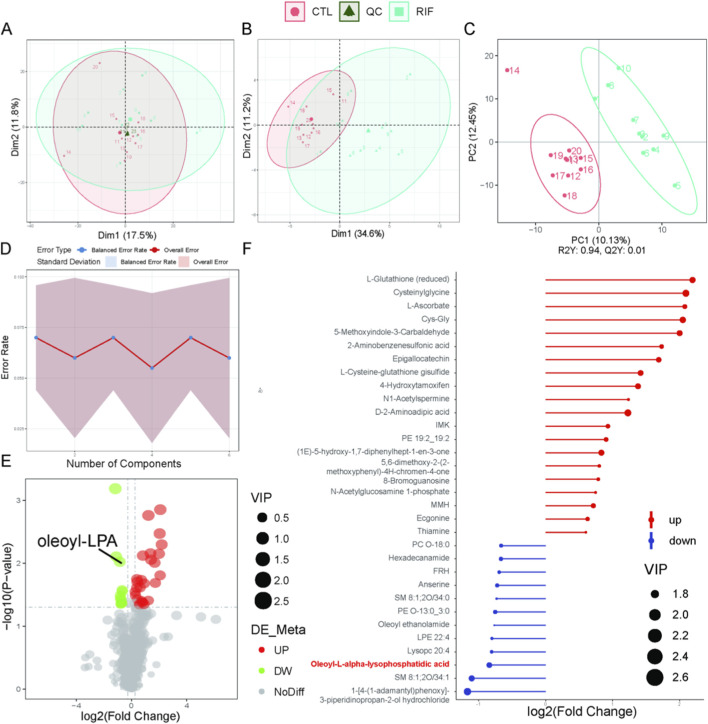
Metabolomics analysis in positive ion mode. **(A)** Principal component analysis (PCA) scatter plot of raw metabolomics data with quality control (QC) samples. **(B)** PCA scatter plot of differentially expressed metabolites (DEMs). **(C)** Partial least squares-discriminant analysis (PLS-DA) score plot of the metabolome. For **(A–C)**: Red represents the control group; light blue represents the recurrent implantation failure (RIF) group; dark green represents QC samples. **(D)** Permutation test of PLS-DA. **(E)** Volcano plot of DEMs. Red indicates significantly increased metabolites; green indicates significantly decreased metabolites. **(F)** Stick plot of DEMs (n = 10 per group).

**FIGURE 2:**
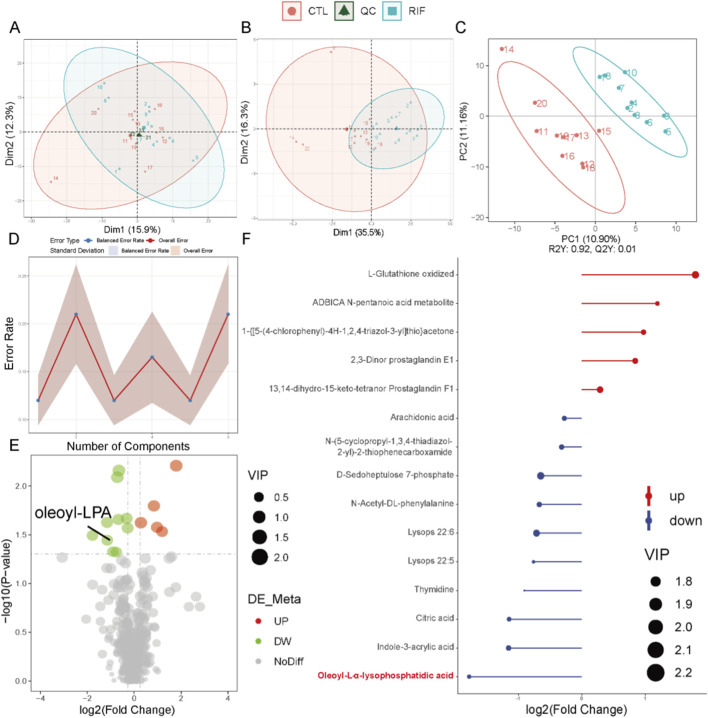
Metabolomics analysis in negative ion mode. **(A)** Principal component analysis (PCA) scatter plot of raw metabolomics data with quality control (QC) samples. **(B)** PCA scatter plot of differentially expressed metabolites (DEMs). **(C)** Partial least squares-discriminant analysis (PLS-DA) score plot of the metabolome. For **(A–C)**: Red represents the control group; light blue represents the recurrent implantation failure (RIF) group; dark green represents QC samples. **(D)** Permutation test of PLS-DA. **(E)** Volcano plot of DEMs. Red indicates significantly increased metabolites; green indicates significantly decreased metabolites. **(F)** Stick plot of DEMs (n = 10 per group).

In both positive and negative ion modes, the PCA score plots of quality control (QC) samples (Figures 1A, 2A) showed tight clustering, indicating high instrumental stability during data acquisition. Based on the diagnostic criteria described in the Methods section, 39 DEMs were identified in positive ion mode and 15 in negative ion mode. PCA score plots revealed significantly distinct metabolic profiles between the RIF group and the control group (Figures 1B, 2B). The PLS-DA score plots further demonstrated clear separation and clustering patterns between the two groups (Figures 1C, 2C). Permutation test results (Figures 1D, 2D) confirmed the robustness of the PLS-DA model, with a permutation-derived variability of less than 0.25, indicating that the model was not overfitted and had strong predictive capability. Expression patterns of the DEMs in the RIF and control groups were visualized (Figures 1E, 2E) and summarized in Supplementary Table S3, showing that in positive ion mode, 27 metabolites were upregulated and 12 were downregulated, while in negative ion mode, 5 were upregulated and 10 were downregulated. Notably, lysophosphatidic acid (LPA) was identified as a differentially abundant metabolite in both ion modes (Figures 1F, 2F). In positive ion mode, LPA had a FC value of 0.56 and a *p*-value of 0.009; in negative ion mode, its FC value was 0.29 with a *p*-value of 0.032.

To further validate the reduced level of LPA observed in the RIF group, we examined the mRNA expression of key enzymes involved in LPA synthesis (ATX, PLA1, and PLA2) and degradation (PPAP2A, PPAP2B, and PPAP2C) in endometrial tissues from both control and RIF groups. The results showed that ATX and PLA1, genes associated with LPA synthesis, were slightly downregulated in the RIF group, although not significantly. In contrast, PPAP2A involved in LPA degradation was slightly upregulated without statistical significance, while PPAP2B and PPAP2C were significantly upregulated in RIF (Supplementary Figure S1, *p* < 0.05). These findings suggest that the significant decrease in LPA levels in RIF may result from both a moderate reduction in its synthesis and a pronounced enhancement of its degradation.

### 3.2 Expression of LPAR1-6 in the GEO database and clinical samples

**FIGURE 3:**
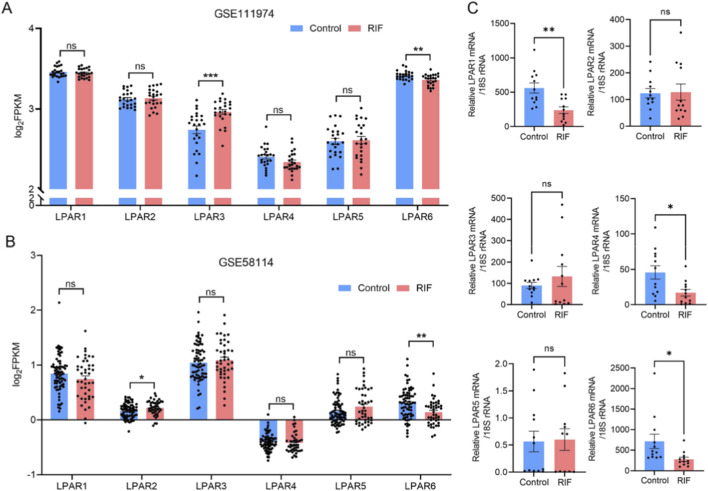
Expression profiles of LPAR1-6 in public databases and clinical specimens. **(A)** Differential expression of LPAR1-6 between control and RIF groups based on dataset GSE111974. **(B)** Differential expression of LPAR1-6 between control and RIF groups based on dataset GSE58144. **(C)** Validation of LPAR1–6 expression in clinical endometrial samples via RT-qPCR (n = 12 per group). ***, *p* < 0.001; **, *p* < 0.01; *, *p* < 0.05; ns, not significant. Blue bars indicate the control group; red bars indicate the RIF group.

To characterize the expression profiles of LPA receptor (LPAR) family members (LPAR1-6), we analyzed human endometrial transcriptome data from the GEO database, selecting datasets with sample sizes ≥20 that included both women with RIF and healthy controls. The datasets GSE111974 (24 RIF and 24 controls) and GSE58144 (43 RIF and 72 controls) were retrieved to evaluate LPAR expression. Analysis revealed that LPAR3 was significantly upregulated and LPAR6 significantly downregulated in RIF compared with controls in GSE111974, while in GSE58144, LPAR2 was significantly upregulated and LPAR6 significantly downregulated (Figures 3A,B). We further validated these findings using RT-qPCR on endometrial tissues from 12 RIF patients and 12 fertile controls, which demonstrated significant downregulation of LPAR1, LPAR5, and LPAR6 in the RIF group (Figure 3C).

Given the potential indirect influence of male-factor infertility on endometrial receptivity, we additionally examined the expression of key endometrial functional markers—FOXO1, HAND2, HOXA10, and KI67—in both groups. Immunohistochemistry results indicated that HOXA10 was significantly downregulated (*p* < 0.05) and KI67 significantly upregulated (*p* < 0.01) in the RIF group compared to controls. FOXO1 and HAND2 also exhibited downward trends, although these changes were not statistically significant (*p* > 0.05) (Supplementary Figure S2A). Consistent with these observations, Western blot analysis showed marked downregulation of HAND2 (*p* = 0.002) and non-significant decreasing trends for HOXA10 (*p* = 0.057) and FOXO1 (*p* > 0.05) in the RIF group (Supplementary Figure S2B).

### 3.3 LPA promotes decidualization of hESCs via LPARs

**FIGURE 4:**
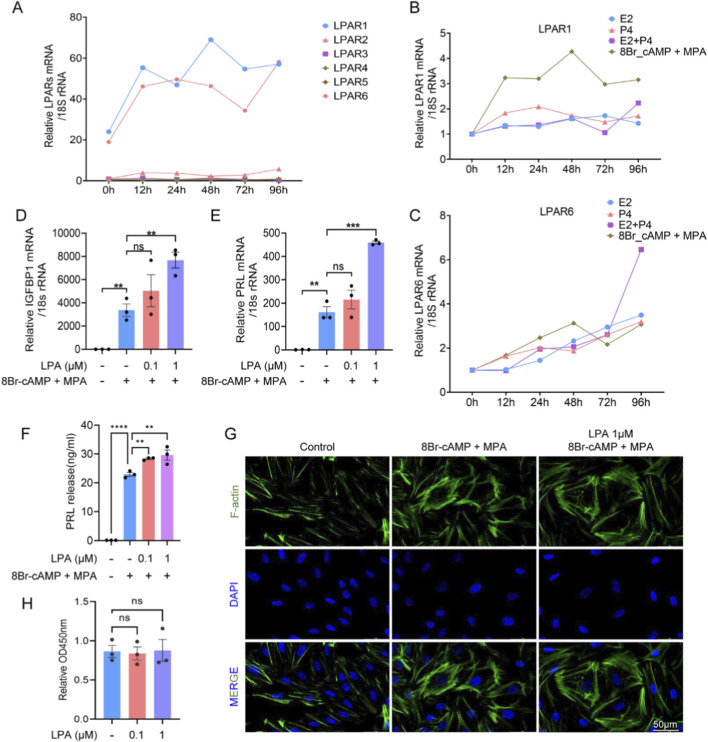
LPA promotes decidualization in human endometrial stromal cells (hESCs). **(A)** Expression of LPAR1-6 during hESC decidualization at indicated time points. **(B,C)** Time-dependent LPAR1 **(B)** and LPAR6 **(C)** expression under E_2_, P_4_, E_2_+P_4_, or 8-Br-cAMP + MPA treatment. **(D,E)** mRNA levels of IGFBP1 **(D)** and PRL **(E)** in hESCs treated with LPA during decidualization. **(F)** PRL protein secretion measured by ELISA. **(G)** Cytoskeletal reorganization visualized by immunofluorescence. **(H)** Cell proliferation assessed by CCK-8 assay. Error bars indicate SEM; data represent mean ± SEM from ≥3 experiments. ****, *p* < 0.0001; ***, *p* < 0.001; **, *p* < 0.01; ns, not significant.

Endometrial stromal cells, as the primary cellular constituents of the endometrium, play essential roles in decidualization. To ensure high purity and minimal epithelial contamination, stromal cells were isolated from fresh endometrial tissues obtained from fertile control women. The isolated cells were characterized using flow cytometry and immunohistochemistry for the stromal marker vimentin and the epithelial marker cytokeratin. Results demonstrated a stromal cell purity of 98.54%, as indicated by the proportion of vimentin-positive/cytokeratin-negative cells (Supplementary Figure S3), confirming the high quality of the isolated cell population for subsequent experiments. Given the function of ESCs as receptors of LPA, the relative expression of LPAR1-6 during decidualization was measured in hESCs by RT-qPCR. LPAR1 and LPAR6 were highly expressed throughout the decidualization process (Figure 4A), and consequently, the effects of E2 and P4 on LPAR1 and LPAR6 regulation were determined. Primary cultured hESCs were treated with E2, P4, E2+P4, 8Br-cAMP + MPA, and cells were harvested at various time points (Figures 4B,C). In addition, the impact of LPA on decidualization was evaluated by incubating hESCs with a range of LPA concentrations. LPA treatment (1 μM) significantly increased the mRNA levels of IGFBP1 and PRL, and the PRL protein level (Figures 4D–F). F-actin staining demonstrated the anticipated cytoskeletal reorganization and morphological changes, consistent with the transition from a fibroblast-like to a decidual phenotype. Specifically, the cells changed from long, spindle-shaped forms to larger, more rounded shapes (Figure 4G). These results collectively demonstrated that LPA promoted the decidualization of hESCs at the mRNA, protein, and morphological levels. LPA had no discernible influence on cell proliferation (Figure 4H).

### 3.4 Inhibition of LPAR6, but not LPAR1, attenuates decidualization of hESCs

**FIGURE 5:**
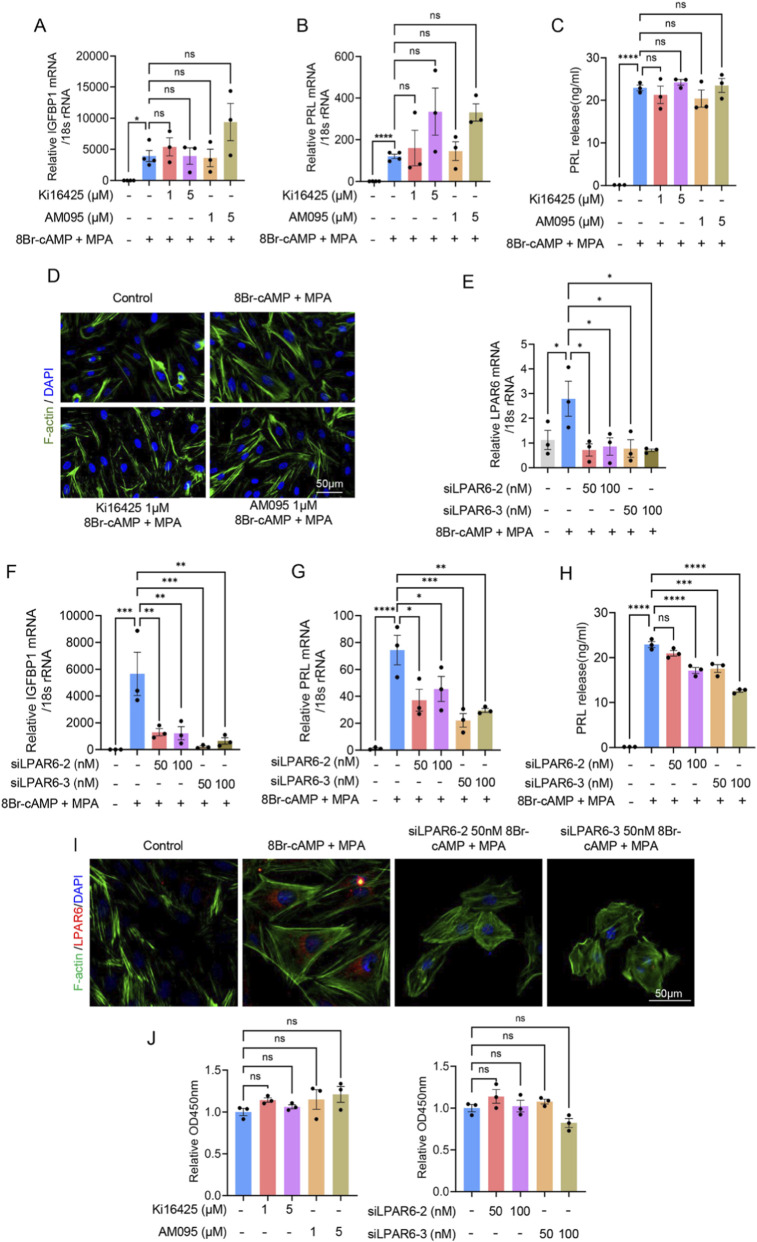
Inhibition of LPAR6, but not LPAR1, attenuates decidualization of hESCs. **(A)** RT-qPCR showing relative IGFBP1 mRNA level in hESCs during decidualization after adding LPAR1 antagonists, AM095 or Ki16425. **(B)** RT-qPCR showing relative PRL mRNA level in hESCs during decidualization after adding LPAR1 antagonists, AM095 or Ki16425. **(C)** ELISA showing relative PRL protein level in hESCs during decidualization after adding LPAR1 antagonists, AM095 or Ki16425. **(D)** Immunofluorescence showing changes in cytoskeletal morphology in hESCs during decidualization after adding LPAR1 antagonists, AM095 or Ki16425. **(E)** The efficiency of LPAR6 knockdown by siLPAR6-2/3 detected with RT-qPCR. **(F)** RT-qPCR showing relative IGFBP1 mRNA level in hESCs during decidualization after adding siLPAR6. **(G)** RT-qPCR showing relative PRL mRNA level in hESCs during decidualization after adding siLPAR6. **(H)** ELISA showing relative PRL protein level in hESCs during decidualization after adding siLPAR6. **(I)** Immunofluorescence showing changes in cytoskeletal morphology in hESCs during decidualization after adding siLPAR6. **(J)** CCK-8 assay showing the effect of LPAR1 antagonists or siLPAR6 on cell proliferation. Error bars represent SEM, and the data are means of at least three independent experiments. ****, *p* < 0.0001; ***, *p* < 0.001; **, *p* < 0.01; *, *p* < 0.05; ns, not significant.

To determine whether LPAR1 or LPAR6 was involved in regulating decidualization, we inhibited LPAR1 in hESCs using pharmacological antagonists (AM095 or Ki16425) and knocked down LPAR6 expression with specific siRNAs (siLPAR6-2/3). Inhibition of LPAR1 did not significantly alter the mRNA levels of IGFBP1 and PRL, nor affect PRL protein secretion (Figures 5A–C). Furthermore, LPAR1 blockade had no observable effect on cytoskeletal organization, as assessed by F-actin staining (Figure 5D). In contrast, LPAR6 knockdown (validated by RT-qPCR; Figure 5E) markedly reduced IGFBP1 and PRL mRNA expression, decreased PRL protein secretion, and disrupted cytoskeletal morphology (Figures 5F–I). These results indicate that LPAR6, but not LPAR1, is critical for decidualization, influencing transcriptional, translational, and structural aspects of the process. Additionally, neither LPAR1 inhibition nor LPAR6 knockdown affected cell proliferation (Figure 5J).

### 3.5 LPAR6 is involved in LPA-mediated regulation of decidualization

**FIGURE 6:**
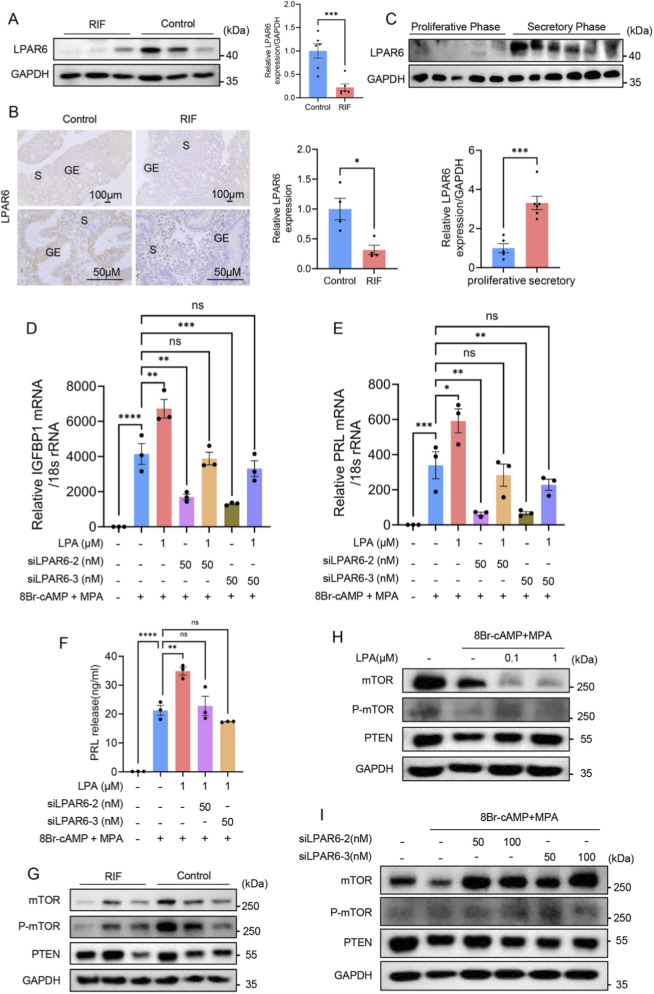
LPA affects decidualization of hESCs by regulating LPAR6. **(A)** Expression of LPAR6 protein between RIF and control groups detected by Western blotting (n = 6 per group). **(B)** Expression of LPAR6 protein between RIF and control groups detected by immunohistochemical staining (n = 4 per group). **(C)** The protein expression of LPAR6 between proliferative (n = 5) and secretory phases (n = 6) detected by Western blotting. **(D)** RT-qPCR showing relative IGFBP1 mRNA level in hESCs during decidualization after adding LPA and siLPAR6. **(E)** RT-qPCR showing relative PRL mRNA level in hESCs during decidualization after adding LPA and siLPAR6. **(F)** ELISA showing relative PRL protein level in hESCs during decidualization after adding LPA and siLPAR6. **(G)** Western blotting showing protein levels of mTOR, P-mTOR, and PTEN between RIF and control groups (n = 3 per group). **(H)** Western blotting showing protein levels of mTOR, P-mTOR, and PTEN after the administration of LPA. **(I)** Western blotting showing protein levels of mTOR, P-mTOR, and PTEN after adding siLPAR6. ****, *p* < 0.0001; ***, *p* < 0.001; **, *p* < 0.01; *, *p* < 0.05; ns, not significant.

Based on the above results, we determined the expression of LPAR6 protein in clinical samples from RIF and control groups and also made comparisons between the proliferative and secretory phases. Western blotting and immunohistochemical staining revealed that the protein expression of LPAR6 was lower in the endometrium of RIF patients compared with controls (Figures 6A,B). Moreover, the protein expression of LPAR6 was higher in the secretory phase than in the proliferative phase (Figure 6C). Further experiments in hESCs demonstrated that after the inhibition of LPAR6, LPA no longer had an effect on the mRNA levels of IGFBP1 and PRL, or on PRL protein level (Figures 6D–F). It is worth noting that the addition of LPA after inhibition of LPAR6 did not rescue the expression of IGFBP1 and PRL compared with the sole inhibition of LPAR6 (Figures 6D,E). These results together support our hypothesis that LPAR6 is involved in the LPA-mediated regulation of decidualization.

Next, we attempted to identify the downstream signaling pathways that could mediate decidualization in clinical samples and hESCs. There were no significant differences in protein levels of mTOR, P-mTOR, and PTEN between RIF and control groups, which may be due to the limited sample size (Figure 6G). The Western blotting results showed that LPA had no evident influence on the expression of P-mTOR and PTEN but substantially reduced the expression of mTOR (Figure 6H). The inhibition of LPAR6 also had no effect on PTEN expression, but it did unexpectedly upregulate the expression of mTOR and P-mTOR (Figure 6I).

## 4 Discussion

Decidualization is essential for the establishment of endometrial receptivity and successful embryo implantation. Our findings are grounded in an integrative multi-omics approach. Metabolomic analysis of endometrial tissues from women with RIF and controls revealed significantly altered LPA levels, supported by dysregulated expression of LPA metabolic enzymes. Transcriptomic data from public GEO databases and clinical samples further indicated abnormal expression of LPA receptors, particularly LPAR6, which was consistently downregulated in RIF endometria. Thus, we employed an *in vitro* model of decidualization in hESCs using MPA and 8Br-cAMP. The process was confirmed by elevated expression of decidual markers IGFBP1 and PRL, along with characteristic morphological changes. Notably, we found that LPA enhanced decidualization, as evidenced by increased IGFBP1 and PRL mRNA levels and elevated PRL secretion. These results not only confirm the role of LPA in decidualization but also provide novel insights into the metabolic and molecular disturbances underlying RIF, highlighting the potential targeting of the LPA-LPAR6 axis in therapeutic strategies for improving endometrial receptivity.

LPAs are ubiquitous bioactive phospholipids derived from membrane phospholipid metabolism by autotaxin (ATX) (Choi et al., 2010). Structurally characterized by a glycerol backbone, a phosphate group, and a long fatty acyl chain (Lin et al., 2020), LPA can be generated intracellularly and extracellularly. Extracellular production occurs mainly through two pathways: hydrolysis of lysophospholipids (LPLs) by ATX following PLA1/PLA2 activity, or conversion of phosphatidic acid (PA) to LPA via membrane-associated PA-selective phospholipases A1 (Richmond and Smith, 2011; Aoki et al., 2008). Extracellular LPA is primarily degraded by lipid phosphate phosphatases (LPPs/PPAP2 family) (Jose et al., 2024). As an extracellular signaling molecule, LPA regulates diverse physiological and pathological processes including nervous system development, hematopoiesis, tumor progression, and reproduction (Lin et al., 2020; Ye and Chun, 2010; Birgbauer, 2021; Balijepalli et al., 2021). Previous studies implicated LPA in pregnancy maintenance (Tokumura et al., 2002), embryo expansion (Shiokawa et al., 2000), and uterine contractility (Nagashima et al., 2023). In the present study, metabolomic profiling revealed, for the first time, significantly reduced LPA levels in the endometrium of patients with RIF. Functional experiments demonstrated that oleoyl-LPA upregulated the decidual markers IGFBP1 and PRL and induced cytoskeletal remodeling in hESCs, supporting a promotive role of LPA in decidualization.

LPA signals through 6 G protein-coupled receptors (LPAR1-6) to modulate multiple reproductive processes including fertilization, decidualization, implantation, and pregnancy maintenance (Ye and Chun, 2010). For example, LPAR1 activation induces IL-8 via NF-κB, promoting endometrial angiogenesis (Chen et al., 2008), while disrupted LPA-LPAR signaling increases miscarriage rates in mice (Yang et al., 2022). LPAR3 is critical for embryo spacing and implantation (Hama et al., 2007; Ye et al., 2005) and facilitates vascular remodeling at the maternal-fetal interface (Sordelli et al., 2017). Our multi-dataset bioinformatic analysis revealed aberrant expression of LPAR2, LPAR3, and LPAR6 in RIF. Clinical validation further confirmed dysregulation of LPAR1, LPAR5, and LPAR6 in RIF endometria. Based on their high expression during *in vitro* decidualization, we focused on LPAR1 and LPAR6. Functional studies showed that only LPAR6 knockdown and not LPAR1 inhibition compromised decidualization, as evidenced by suppressed IGFBP1/PRL expression and disrupted cytoskeletal organization. Although LPAR6 remains understudied, its involvement in cancer progression and survival (Lei et al., 2022; He et al., 2021; Gnocchi et al., 2019) and early pregnancy adaptation in animals (Sadam et al., 2017; Piotrowska-Tomala et al., 2024) highlights its biological significance. Our results establish a critical role for LPAR6 in human decidualization and RIF pathogenesis. We further demonstrated that LPAR6 protein expression is reduced in RIF endometria and elevated during the secretory phase, consistent with a functional role in receptivity. Rescue experiments confirmed that LPA failed to promote decidualization in LPAR6-inhibited hESCs, indicating that LPAR6 is the primary receptor mediating LPA’s effects during this process.

To explore downstream mechanisms, we evaluated the PTEN/mTOR pathway, which is central to decidualization. While PTEN, a negative regulator of PI3K, is typically upregulated in decidualization (Li et al., 2022), we observed no significant change in response to LPA or LPAR6 inhibition. In contrast, LPA downregulated mTOR expression, whereas LPAR6 inhibition increased both mTOR and phospho-mTOR levels. These seemingly discordant results suggest context-dependent crosstalk between LPA-LPAR6 signaling and mTOR activity, warranting further investigation to elucidate the precise regulatory network.

Several limitations of this study should be acknowledged. First, the sample size used in the metabolomic analysis was relatively limited, which may have constrained the identification of additional meaningful metabolites and reduced the robustness of the findings against individual variations. Second, the *in vitro* hESC model, while informative, does not fully recapitulate the complex *in vivo* endometrial microenvironment, particularly the interactions with immune cells, endothelial cells, and epithelial components. Therefore, the conclusions drawn from this simplified model require further validation in more physiologically relevant systems. Finally, *in vivo* studies using LPAR6 knockout mice or established RIF animal models are necessary to confirm the physiological relevance and therapeutic potential of targeting the LPA-LPAR6 axis. Despite these limitations, our findings provide novel insights into the metabolic and molecular mechanisms underlying RIF and establish a foundation for future functional and translational studies.

In conclusion, our integrated metabolomic-transcriptomic-functional approach revealed that LPA was significantly downregulated in the endometrium of RIF patients, and identified the dysregulated LPA-LPAR6 signaling axis as a key contributor to impaired decidualization. We further demonstrated that both LPAR1 and LPAR6 were highly expressed during decidualization of hESCs, but functional experiments established that LPA promoted decidualization through LPAR6 rather than LPAR1. These findings provide new mechanistic insights into the pathogenesis of RIF and highlight LPAR6 as a promising therapeutic target for improving endometrial receptivity in affected patients.

## Data Availability

The original contributions presented in the study are included in the article/Supplementary Material, further inquiries can be directed to the corresponding authors.

## Abbreviations

ART, assisted reproductive technology; ATX, autotaxin; DEMs, differentially expressed metabolites; ELISA, enzyme-linked immunosorbent assay; FC, fold change; hESCs, human endometrial stromal cells; LPA, lysophosphatidic acid; LPAR, lysophosphatidic acid receptor; LPLs, lysophospholipids; MPA, medroxyprogesterone acetate; PA, phosphatidic acid; PLS-DA, partial least squares discriminant analysis; QC, quality control; RIF, recurrent implantation failure; RT-qPCR, Real-time quantitative PCR; siRNAs, small interfering RNAs; VIP, variable importance value.
