# Supplementary material for: Lysophosphatidic acid promotes endometrial decidualization in recurrent implantation failure patients by regulating LPAR6
Source: Front Cell Dev Biol. 2025 Oct 22;13:1652740. doi: 10.3389/fcell.2025.1652740 (PMC12586045; doi:10.3389/fcell.2025.1652740)
Supplement: Supplementary file 1 [file Table1.docx]

**Supplementary Materials**


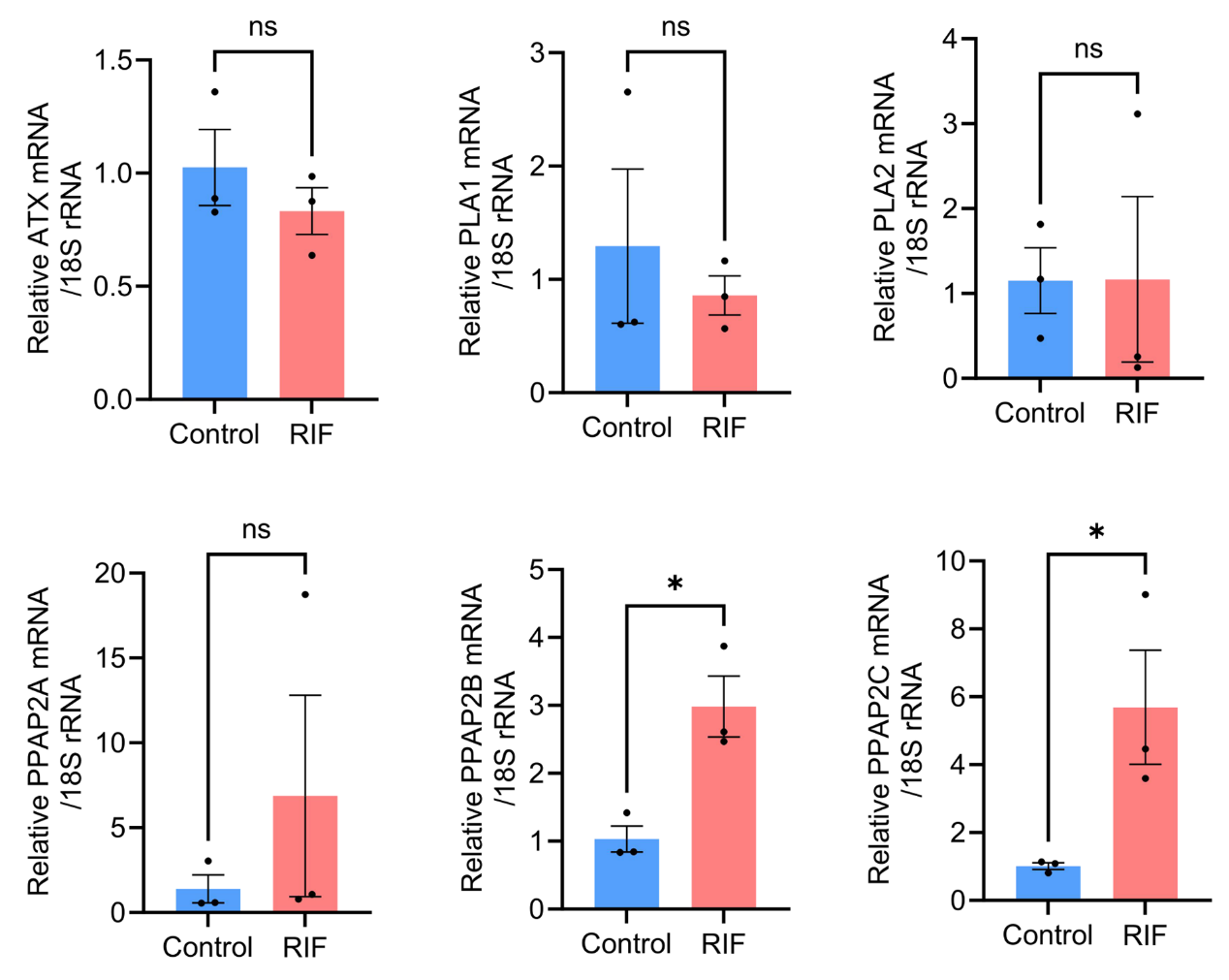


**Figure S1.** Expression of genes encoding enzymes involved in LPA synthesis (ATX, PLA1, PLA2) and degradation (PPAP2A, PPAP2B, PPAP2C) in endometrial tissues from control and RIF groups (n = 3 per group). Blue bars represent the control group; red bars represent the RIF group. *, *p* < 0.05; ns, not significant.


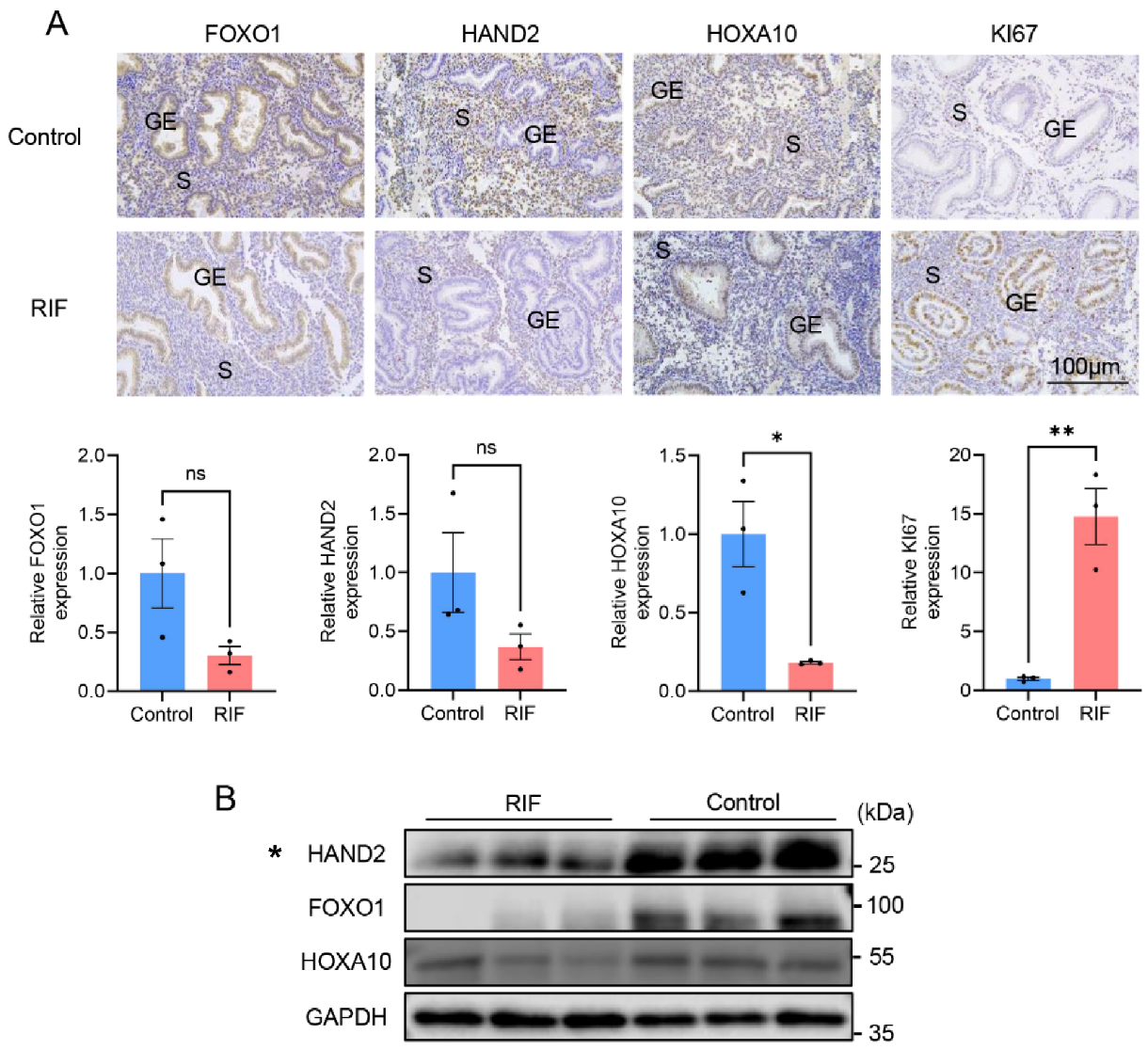


**Figure S2.** Expression of endometrial receptivity markers between the control and RIF groups (n = 3 per group). ***p* <0.01, **p* <0.05; ns, not significant.


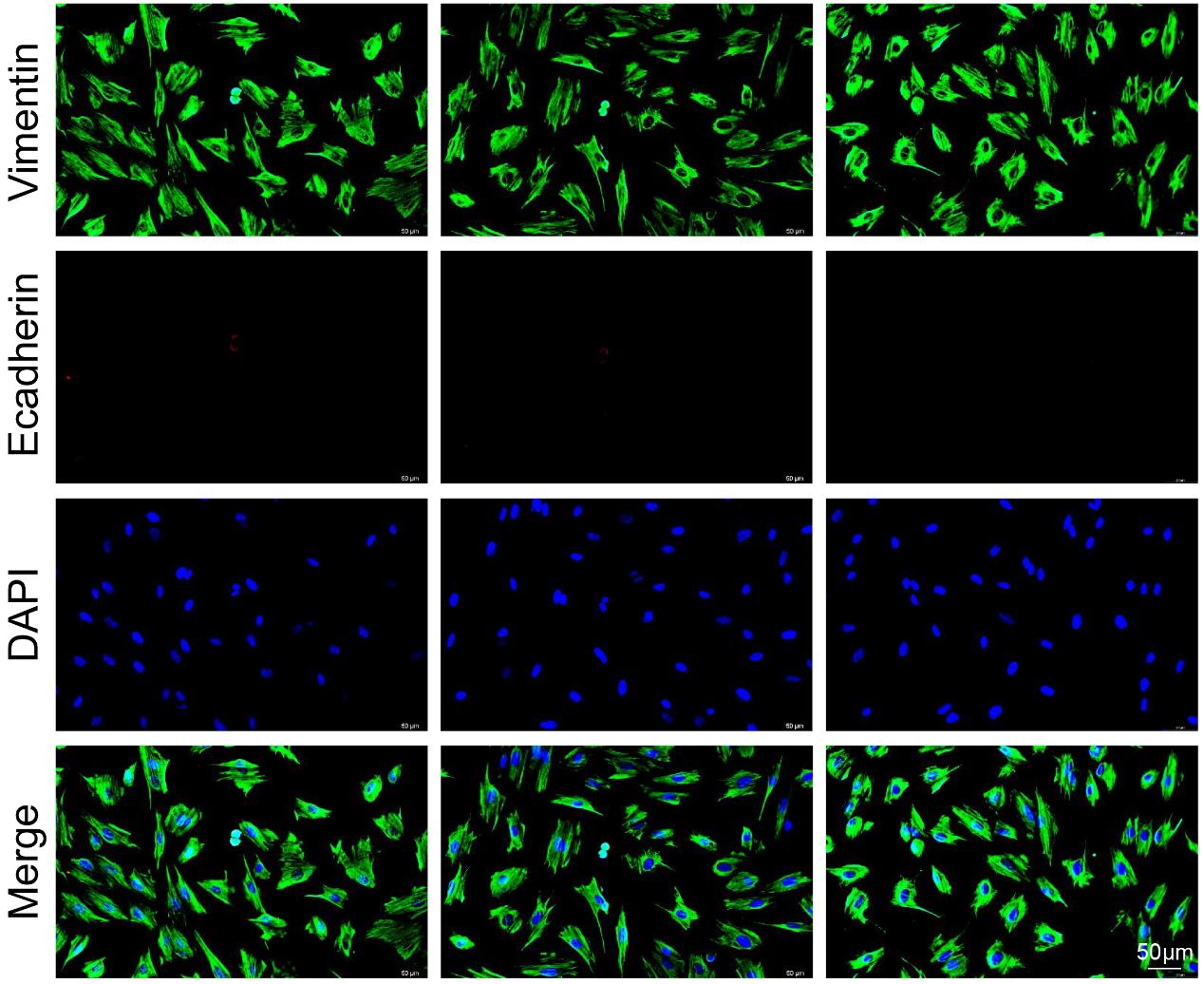


**Figure S3.** Purity of endometrial stromal cells isolated from fresh endometrial tissues of fertile control women.

**Table S1.** Primer sequences used in this study.

| **Gene** | **Forward primer** | **Reverse primer** |
| --- | --- | --- |
| h18s | CGGCTACCACATCCAAGGAA | CTGGAATTACCGCGGCT |
| IGFBP1 | TTTTACCTGCCAAACTGCAACA | CCCATTCCAAGGGTAGACGC |
| PRL | GGAGCAAGCCCAACAGATGAA | GGCTCATTCCAGGATCGCAAT |
| LPAR1 | GGGCCTCATTGACACCAGC | GGAAAACCGTAATGTGCCTCTC |
| LPAR2 | CCTGGTCAAGACTGTTGTCATC | GACTCACAGCCTAAACCATCC |
| LPAR3 | GCTGCCGATTTCTTCGCTG | AGCAGTCAAGCTACTGTCCAG |
| LPAR4 | GCAAGCCTGCTACTCTGTCTC | CCAGGGCATACAAGAAGAGGAC |
| LPAR5 | CACTTGGTGGTCTACAGCTTG | GCGTAGTAGGAGAGACGAACG |
| LPAR6 | GGACAATGTACCCAATCACTCTC | ACTTCTCCTGACAGACCAGTTT |
| ATX | ACTTTTGCCGTTGGAGTCAAT | GGAGTCTGATAGCACTGTAGGA |
| PLA1 | AGTGGGGATGTAGCCGATTC | GCCTGTTGAAATCTAGGCCAT |
| PLA2 | ATGAAGACCCTCCTACTGTTGG | GCTTCCTTTCCTGTCGTCAACT |
| PPAP2A | GGCAGGTTGTCCTTCTATTCAG | CAGTGTGGGGCGTAAGAGT |
| PPAP2B | TGAGAGCATCAAGTACCCACT | ACGTAGGGGTTCTGAATCGTC |
| PPAP2C | TCTATTCTCGCTCGGACTTCA | TCAGACGCCCAATCATGTACT |

**Table S2.** Antibodies and reagents used in this study.

| **Antibody** | **Application (dilution)** | **Source** | **Catalogue number** |
| --- | --- | --- | --- |
| GAPDH | WB (1:10000) | Bioworld Technology | AP0063 |
| Phalloidin-iFluor 488 | IF (1:1000) | Abcam | ab176753 |
| DAPI | IF (1:1000) | Servicebio | G1012 |
| Alexa Fluor 594-conjugated secondary antibody | IF (1:1000) | Abcam | ab150080 |
| HRP-conjugated anti-rabbit IgG | WB (1:10000), IHC (1:1000) | Cell Signaling Technology | 7074S |
| HRP-conjugated anti-mouse IgG | WB (1:10000) | Cell Signaling Technology | 7076S |
| LPAR6 | IHC (1:300) | Invitrogen | PA5-115705 |
| LPAR6 | WB (1:1000) | CUSABIO | PA017336 |
| mTOR | WB (1:1000) | Proteintech | 66888-1 |
| p-mTOR | WB (1:1000) | Proteintech | 67778-1 |
| PTEN | WB (1:1000) | Proteintech | 22034-1 |
| KI67 | IHC (1:1000) | Abcam | ab16667 |
| HOXA10 | IHC (1:500) | Proteintech | 26497-1 |
| HOXA10 | WB (1:1000) | Proteintech | 26497-1 |
| FOXO1 | IHC (1:500) | Cell Signaling Technology | 2880T |
| FOXO1 | WB (1:1000) | Cell Signaling Technology | 2880T |
| HAND2 | IHC (1:500) | Abcam | ab200040 |
| HAND2 | WB (1:1000) | Abcam | ab200040 |
| Vimentin | IF (1:500) | Abcam | ab92547 |
| Ecadherin | IF (1:200) | Invitrogen | 14-3249-82 |

**Table S3.** The differential analysis information on the metabolomics.

| **Metabolites** | **Ion mode** | **FC** | **log2FC** | **P-value** | **VIP** |
| --- | --- | --- | --- | --- | --- |
| 1-[4-(1-adamantyl)phenoxy]-3-piperidinopropan-2-ol hydrochloride | Positive | 0.445 | -1.168 | 0.001 | 2.633 |
| Cysteinylglycine | Positive | 4.268 | 2.094 | 0.001 | 2.496 |
| D-2-Aminoadipic acid | Positive | 2.342 | 1.228 | 0.002 | 2.476 |
| Cys-Gly | Positive | 4.132 | 2.047 | 0.003 | 2.339 |
| L-Glutathione (reduced) | Positive | 4.570 | 2.192 | 0.005 | 2.293 |
| 4-Hydroxytamoxifen | Positive | 2.602 | 1.379 | 0.007 | 2.206 |
| 5-Methoxyindole-3-Carbaldehyde | Positive | 3.999 | 2.000 | 0.007 | 2.223 |
| (1E)-5-hydroxy-1,7-diphenylhept-1-en-3-one | Positive | 1.780 | 0.831 | 0.008 | 2.251 |
| SM 8:1;2O/34:1 | Positive | 0.465 | -1.105 | 0.008 | 2.258 |
| L-Cysteine-glutathione disulfide | Positive | 2.672 | 1.418 | 0.009 | 2.187 |
| Oleoyl-L-alpha-lysophosphatidic acid | Positive | 0.558 | -0.842 | 0.010 | 2.170 |
| Epigallocatechin | Positive | 3.221 | 1.687 | 0.010 | 2.116 |
| L-Ascorbate | Positive | 4.220 | 2.077 | 0.016 | 2.003 |
| Taurine | Positive | 1.317 | 0.397 | 0.018 | 1.997 |
| MMH | Positive | 1.637 | 0.711 | 0.018 | 2.028 |
| Benzoic acid | Positive | 1.346 | 0.429 | 0.020 | 2.176 |
| 2-Aminobenzenesulfonic acid | Positive | 3.317 | 1.730 | 0.021 | 1.964 |
| IMK | Positive | 1.905 | 0.929 | 0.021 | 1.946 |
| Ecgonine | Positive | 1.543 | 0.626 | 0.024 | 1.922 |
| N-Acetylmethionine | Positive | 1.305 | 0.384 | 0.025 | 1.980 |
| PC O-18:0 | Positive | 0.631 | -0.665 | 0.027 | 1.866 |
| Hexadecanamide | Positive | 0.629 | -0.668 | 0.028 | 1.900 |
| Nicotinamide | Positive | 1.205 | 0.269 | 0.029 | 1.839 |
| 5-Hydroxymethyluracil | Positive | 1.226 | 0.294 | 0.032 | 1.855 |
| LysoPC 20:4 | Positive | 0.572 | -0.807 | 0.032 | 1.813 |
| LPE 22:4 | Positive | 0.573 | -0.802 | 0.036 | 1.782 |
| 3-(allylsulfanyl)-4-[4-(benzyloxy)phenyl]-5-methyl-4H-1,2,4-triazole | Positive | 1.356 | 0.439 | 0.037 | 1.872 |
| PE O-13:0_3:0 | Positive | 0.593 | -0.753 | 0.037 | 1.912 |
| N1-Acetylspermine | Positive | 2.355 | 1.236 | 0.039 | 1.764 |
| N-Acetylglucosamine 1-phosphate | Positive | 1.675 | 0.744 | 0.040 | 1.756 |
| 5,6-dimethoxy-2-(2-methoxyphenyl)-4H-chromen-4-one | Positive | 1.742 | 0.800 | 0.042 | 1.809 |
| 8-Bromoguanosine | Positive | 1.723 | 0.785 | 0.042 | 1.830 |
| FRH | Positive | 0.618 | -0.694 | 0.043 | 1.820 |
| Anserine | Positive | 0.605 | -0.724 | 0.043 | 1.879 |
| SM 8:1;2O/34:0 | Positive | 0.602 | -0.731 | 0.043 | 1.738 |
| PE 19:2_19:2 | Positive | 1.869 | 0.902 | 0.045 | 1.974 |
| Oleoyl ethanolamide | Positive | 0.587 | -0.767 | 0.045 | 1.720 |
| gamma-glutamylglutamine | Positive | 1.468 | 0.554 | 0.045 | 1.762 |
| Thiamine | Positive | 1.515 | 0.600 | 0.046 | 1.739 |
| L-Glutathione (oxidized) | Negative | 3.459 | 1.790 | 0.006 | 2.283 |
| D-Sedoheptulose 7-phosphate | Negative | 0.638 | -0.648 | 0.007 | 2.281 |
| LysoPS 22:6 | Negative | 0.609 | -0.715 | 0.008 | 2.198 |
| 2,3-Dinor prostaglandin E1 | Negative | 1.792 | 0.842 | 0.016 | 2.024 |
| N-(5-cyclopropyl-1,3,4-thiadiazol-2-yl)-2-thiophenecarboxamide | Negative | 0.803 | -0.317 | 0.021 | 1.942 |
| N-acetyl-DL-phenylalanine | Negative | 0.628 | -0.671 | 0.022 | 1.947 |
| Indole-3-acrylic acid | Negative | 0.449 | -1.155 | 0.024 | 1.954 |
| 13,14-dihydro-15-keto-tetranor prostaglandin F1β | Negative | 1.221 | 0.288 | 0.024 | 2.129 |
| 1-{[5-(4-chlorophenyl)-4H-1,2,4-triazol-3-yl]thio}acetone | Negative | 1.962 | 0.972 | 0.026 | 1.946 |
| Arachidonic acid | Negative | 0.827 | -0.275 | 0.027 | 1.908 |
| ADBICA N-pentanoic acid metabolite | Negative | 2.283 | 1.191 | 0.029 | 1.858 |
| Oleoyl-L-α-lysophosphatidic acid | Negative | 0.292 | -1.776 | 0.032 | 1.831 |
| Citric acid | Negative | 0.452 | -1.147 | 0.036 | 1.864 |
| Thymidine | Negative | 0.533 | -0.907 | 0.047 | 1.712 |
| Lysops 22:5 | Negative | 0.590 | -0.762 | 0.048 | 1.762 |
